# Stressful Life Events and Sense of Coherence in College Students: Roles of Coping, Self-Efficacy, and Stress Mindset

**DOI:** 10.3390/bs15060762

**Published:** 2025-06-01

**Authors:** Shuang Yang, Hongyu Ma, Xiangping Zhan

**Affiliations:** 1 Key Laboratory of Adolescent Cyberpsychology and Behavior (CCNU), Ministry of Education, Wuhan 430079, China; ys0225@csust.edu.cn (S.Y.);; 2 Key Laboratory of Human Development and Mental Health of Hubei Province, School of Psychology, Central China Normal University, Wuhan 430079, China; 3 Mental Health Education Center of Changsha University of Science and Technology, Changsha 410114, China

**Keywords:** college students, mental health, stressful life events, sense of coherence, coping style, general self-efficacy, stress mindset

## Abstract

Drawing on Antonovsky’s salutogenic model, this study investigated how stressful life events relate to university students’ sense of coherence (SOC), focusing on the potential mediating roles of coping style and general self-efficacy, and the moderating role of stress mindset. An analysis of data collected from 2454 Chinese college students (63.6% males, 36.4% females) revealed that stressful life events negatively predicted SOC, with coping style and general self-efficacy significantly sequentially mediating this relationship. Furthermore, stress mindset moderated the relationship between stressful life events and coping style, such that a more positive mindset was associated with more adaptive coping under stress. These findings support the dual-pathway structure of the salutogenic model by illustrating both behavioral and perceptual mechanisms. Importantly, they also underscore the idea that stress, when cognitively reappraised and effectively managed, may contribute to the development of SOC—rather than simply undermining it. This highlights the potential value of stress itself within salutogenic processes. The study offers theoretical insights and preliminary directions for strength-based mental health promotion in higher education settings.

## 1. Introduction

The concept of sense of coherence (SOC), comprising three components, comprehensibility (cognitive), manageability (instrumental), and meaningfulness (motivational), is integral to the salutogenic model and the “salutogenic” factors of college students, which describe an individual’s psychological orientation. Individuals possessing strong SOC exhibit pervasive and enduring confidence (Antonovsky, 1987; Super et al., 2015), perceiving the world as comprehensible, manageable, and meaningful. SOC encapsulates one’s perception of life and ability to comprehend stressors, representing a personal mindset characterized by inherent trust. It enables individuals to identify, benefit from, and utilize Generalized Resistance Resources (GRRs) that they possess or can mobilize (Antonovsky, 1987; Eriksson, 2005; Eriksson et al., 2017).

Empirical studies indicate that SOC is a resilience factor that aids individuals in maintaining health during adversity (Kunzler et al., 2020; Schäfer et al., 2022), and that enhancing students’ SOC is crucial for coping with challenges (Kulcar et al., 2023). SOC is also a significant indicator of mental health (Kulcar et al., 2023). Such chronic stressors during college may not immediately manifest as obvious stress symptoms, and their long-term impact on health becomes evident through a decline in SOC (Braun-Lewensohn et al., 2022).

Antonovsky’s (1987) salutogenic model posits that stressors, Generalized Resistance Resources (GRRs), and coping mechanisms are pivotal in shaping the sense of coherence (SOC). Research examining the interplay between stressors and SOC has predominantly focused on the detrimental effects of stressors on SOC. However, the mediating and moderating mechanisms between stressors and SOC have not been explored from the perspective of promoting the development of SOC. This oversight may stem from a lack of comprehensive research on the mechanisms that promote SOC within the framework of the salutogenic model. Therefore, this study aimed to construct a moderated chain mediation model to explore how stressful life events are associated with SOC through various psychological mechanisms, and to examine whether key cognitive factors can buffer or reshape the negative associations typically observed between stressors and SOC.

### 1.1. Relationship Between Stressful Life Events and Sense of Coherence

In the investigation of stressors’ effects on the sense of coherence (SOC), numerous studies have examined the negative predictive impact of major life events on SOC in the investigation of stressor effects on the sense of coherence (SOC) (Lindahl et al., 2021; Peer et al., 2020; Sanna et al., 2022). Antonovsky (1987) identified chronic stressors as life situations, conditions, or characteristics that significantly influence an individual’s life. Chronic resources or stressors in an individual’s environment are prevalent, persistent, and primary determinants of SOC levels. In addition, Braun-Lewensohn and Sagy (2010) and Braun-Lewensohn et al. (2013) during periods of political violence demonstrated that SOC decreased during acute stress situations, but recovered following the resolution of the crisis. However, under chronic stress conditions, the decrease in SOC persisted. Schäfer et al. (2022) observed a decline in SOC levels over time during the pneumonia epidemic, accompanied by an increase in psychopathological symptoms, potentially linked to the level and duration of stressor exposure. Veronese et al. (2022) reported that COVID-19-related stress, burnout, and trauma symptoms were significantly negatively correlated with SOC among healthcare workers. Bachem et al. (2023) found that pandemic-related stress and university stressors were significantly negatively correlated with SOC among Swiss university students. Kulcar et al. (2023) identified a significant decrease in SOC among Australian university students during the pandemic, indicating a compromised mental health. This study included both negative life events and common chronic university stressors. Collectively, these studies provide consistent evidence that stressful life events—particularly chronic or recurring stressors—are negatively associated with SOC across diverse populations and contexts.

### 1.2. The Mediating Role of Coping Style

Both stressors and coping behaviors that individuals adopt are critical determinants of health or illness. Coping styles are intricately related to SOC (Liu et al., 2006), with positive coping styles significantly predicting SOC (H. Y. Liang et al., 2015). An increase in stressful events can lead to a decrease in positive coping and an increase in negative behaviors (S. Yuan et al., 2023; He et al., 2023), which likely results in ineffective stress relief and impacts SOC. SOC involves understanding and flexibly utilizing resources, including replacing the missing resources. The experience of resources and challenges is essential for a robust SOC. Therefore, the utilization of resources (coping) rather than merely possessing them strengthens SOC (Maass et al., 2017). In a clinical trial involving employees aged 31–51 experiencing burnout, Kähönen et al. (2012) suggested that modifying stress responses to facilitate more effective coping can enhance SOC.

Coping has been conceptualized from both trait- and state-oriented perspectives. In the present study, we follow a trait-oriented approach, which views coping as a relatively stable, cross-situational behavioral tendency rooted in personality traits. This perspective is reflected in the Coping Style Scale for Chinese Adults developed by B. Y. Liang and Wu (2013), which distinguishes between problem-focused and emotion-focused coping. These two broad categories are consistent with Lazarus and Folkman (1984) and Lazarus’s (1993) transactional model of stress and coping—one of the most influential frameworks in coping research—where the adaptiveness of coping depends on primary and secondary appraisals of controllability and situational demand.

Rather than labeling coping strategies as inherently positive or negative, the recent literature emphasizes their contextual utility. For example, emotion-focused coping may be particularly effective in uncontrollable situations, while problem-focused coping is more beneficial when the stressor is changeable or solvable. Studies on Chinese university students suggest that emotion-oriented strategies are among the most frequently employed coping methods (J. W. Chen & Wang, 2012), challenging the traditional view that emotion-focused coping is maladaptive. As B. Y. Liang and Wu (2013) argue, coping should be evaluated based on its effectiveness in reducing distress and enhancing well-being within a given context, regardless of its categorical type.

### 1.3. The Chain-Mediating Role of Coping Style and General Self-Efficacy

General self-efficacy pertains to an individual’s comprehensive confidence in their capacity to manage various challenging situations in novel or diverse environments (Schwarzer & Born, 1997). It is derived from past life experiences and significantly influences the perception and evaluation of current and future circumstances. The development of self-efficacy is facilitated by successfully navigating stressors (Antonovsky, 1987). This belief system underpins the action system, offering feedback and shaping beliefs (Antonovsky, 1987). Empirical evidence indicates that positive coping styles are more likely to result in favorable coping experiences, thereby leading to a linear enhancement in self-efficacy (Y. Y. Yuan et al., 2020).

Effectively managing daily stressors and reducing tension can enhance the Sense of Coherence (SOC). Successful tension reduction contributes to improved health, facilitates the acquisition of Generalized Resistance Resources (GRRS), and strengthens SOC (Antonovsky, 1979; Super et al., 2015). GRRS are categorized into internal and external resources. Internal resources encompass cognitive abilities, problem-solving skills, self-efficacy, optimism, self-esteem, narcissism, higher cognitive function, values, and presence and level of self-disturbance. There is a strong positive correlation between SOC and self-efficacy (Greco et al., 2023; Schäfer et al., 2023). As a fundamental internal resource, self-efficacy may reflect the primary content of SOC’s internal resources (Tsuno & Yamazaki, 2007; Zeng et al., 2016). A one-year intervention study involving morbidly obese adults found that baseline self-efficacy scores were significantly correlated with total SOC scores at a 12-month follow-up (Fagermoen et al., 2015). Additionally, a study of college students identified self-efficacy as the most significant factor influencing SOC (Mato & Tsukasaki, 2019).

### 1.4. The Moderating Role of Stress Mindset

Coping behavior, as a response to an individual’s behavioral system in stressful situations, is influenced by the belief system. From the perspective of implicit theory, a positive mindset can enhance psychological, biological, and behavioral outcomes (Yeager & Dweck, 2012). A stress mindset refers to an individual’s belief about the nature of stress—specifically, whether stress is perceived as enhancing or debilitating (Crum et al., 2013). It constitutes a personal cognitive belief and serves as the psychological and motivational foundation for individuals to select and adopt coping behaviors. It shapes how individuals interpret stressful events and affects their emotional and behavioral responses to stress (L. Chen & Qu, 2021; Crum et al., 2013, 2017). Individuals who hold a stress-is-enhancing mindset are more likely to accept challenging situations, adopt adaptive coping strategies, and view stress as an opportunity for growth. Consequently, a stress mindset can function as a moderator that buffers the negative effects of stress and promotes positive coping (Crum et al., 2013; Jamieson et al., 2018). Keech et al. (2018) found that maintaining a stress-is-enhancing mindset enables individuals to take proactive actions under stress and develop more problem-focused behaviors, thereby reducing experiences of depression and improving life satisfaction. Jiang et al. (2019) observed that adolescents with a stress-is-enhancing mindset actively cope with adverse events rather than evade or struggle. Adolescents with this mindset are more likely to accept or utilize stress, and prefer to adjust and manage their goals using stress. In contrast, adolescents with a stress-is-debilitating mindset may evade or struggle with adverse life events, leading to increased stress. Grünenwald et al. (2023) found that possessing a stress-is-enhancing mindset is associated with more frequent use of adaptive coping behaviors, such as positive reframing and active coping.

### 1.5. The Present Study

Previous research on the relationship between stressful life events and sense of coherence (SOC) has mostly treated SOC as a protective factor that operates under stress (Schäfer et al., 2022), or it has focused on how stressors undermine SOC (Lindahl et al., 2021; Sanna et al., 2022). However, Antonovsky’s salutogenic model emphasizes that stressors are not inherently harmful; if effectively managed, they can become catalysts for health promotion. According to this model, successful stress management is the key process that shifts individuals from tension to health. However, most empirical studies have focused on how increasing Generalized Resistance Resources (GRRs) contributes to SOC, while paying limited attention to the potential of stressors to promote health through their interaction with coping processes and GRRs, as emphasized in the salutogenic model.

**Figure 1:**
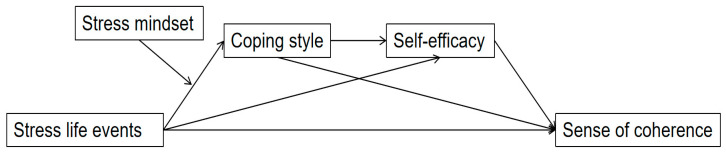
The proposed moderated chain mediation model.

Therefore, the present study aimed to enhance understanding of how stressful life events, coping styles, general self-efficacy, and stress mindset function as key mechanisms in shaping individuals’ sense of coherence. While general self-efficacy was conceptualized as a classical GRR, stress mindset was proposed as a belief-based form of GRR to be empirically tested in this study. Consequently, a moderated chain mediation model was constructed to examine the sequential mediating roles of coping and self-efficacy, as well as the moderating role of stress mindset on the link between stressors and coping behaviors (see Figure 1). The hypotheses were as follows:

**Hypothesis** **1.** *Stressful life events are negatively associated with sense of coherence among college students.*

**Hypothesis** **2.** *Coping style plays a mediating role between stressful life events and sense of coherence.*

**Hypothesis** **3.** *Coping style and general self-efficacy play a chain-mediating role between stressful life events and sense of coherence.*

**Hypothesis** **4.** *Stress mindset moderates the relationship between stressful life events and coping styles.*

## 2. Materials and Methods

### 2.1. Participants

A total of 2454 valid questionnaires were obtained through online convenience sampling from college students across multiple universities in China. Among them, 1560 were male (63.6%) and 894 were female (36.4%). Participants included 1385 freshmen (56.4%), 600 sophomores (24.4%), 375 juniors (15.3%), and 94 seniors (3.8%). Majors covered a range of disciplines including science and engineering (62.1%), humanities and social sciences (28.4%), and others (9.5%).

Given that undergraduate students in China typically fall within a narrow and predictable age range by year level, only academic year was collected and used to reflect age-related differences in this study.

### 2.2. Procedure

Data were collected between March and April 2024 via an anonymous online questionnaire hosted on Wenjuanxing (www.wjx.cn, accessed on 2 April 2024), a widely used Chinese survey platform. The survey link was distributed through university learning platforms and student group messages.

To ensure data quality, responses with completion times under 100 s or duplicated identifiers (e.g., similar IP addresses and demographic patterns) were excluded. A total of 553 invalid responses were removed, yielding 2454 valid questionnaires and an effective response rate of 81.61%.

### 2.3. Ethical Considerations

This study was approved by the Subcommittee for Human Research, Institutional Review Board of the College of Life Sciences, Central China Normal University (CCNU-IRB-202403056A, 28 March 2024), and was conducted in accordance with the Declaration of Helsinki. Participation was anonymous and voluntary. Informed consent was waived in accordance with the ethical standards outlined in the Chinese Guidelines for Ethical Review of Biomedical Research Involving Humans (2020 Revision), which allow for the waiver of written consent in anonymous, minimal-risk social science surveys. All participants were fully informed about the purpose of the study, the voluntary nature of participation, and data confidentiality through an introductory statement at the beginning of the questionnaire. No personal identifying information was collected.

### 2.4. Measurement

#### 2.4.1. Sense of Coherence Scale

The sense of coherence (SOC) was assessed utilizing the 13-item scale (SOC-13) originally developed by Antonovsky (1987) and subsequently revised by Bao and Liu (2005). This scale comprises 13 items, with five items each dedicated to comprehensibility and manageability, and three items focused on meaningfulness. Representative items include “Do you have the feeling that you’re being treated unfairly?” (manageability), “Has it happened in the past that you were surprised by the behavior of people whom you thought you knew?” (comprehensibility), and “Do you have the feeling that you don’t really care about what goes on around you?” (meaningfulness). Each item was rated on a 7-point scale, with five reverse-scored items. The total score was calculated by summing the item scores, with a higher score indicating a stronger sense of coherence. In previous Chinese studies, the SOC-13 has shown good internal consistency (Cronbach’s *α* ranging from 0.78 to 0.89) and a stable three-factor structure. In this study, Cronbach’s *α* for the SOC-13 was 0.858.

#### 2.4.2. Stressful Life Events Scale

Following a comprehensive evaluation involving comparison, pilot testing, and participant interviews, the decision was made to employ the “College Student Stressful Events Scale” developed by Yang (2015). This scale encompasses five dimensions: academic, life, interpersonal, planning, and negative environmental stressors. The items within these dimensions address prevalent sources of stress in college life, encompassing both academic and familial contexts, chronic stressors, and negative life events, with a high likelihood of occurrence. The scale comprises 22 items, each rated on a 5-point Likert scale. Representative items include “Not interested in what you are studying” (academic stressor), “Significant changes in daily life” (life stressor), “Lack of close friends” (interpersonal stressor), “Not good at managing and planning events” (planning stressor), and “Family conflicts” (negative environmental stressor). In the original validation, the scale demonstrated good internal consistency (Cronbach’s *α* = 0.86–0.87), and subscale reliabilities ranged from 0.58 to 0.89. Content and construct validity were also well established. In this study, the items “Experiencing uncontrollable accidents” and “Being robbed or burglarized” were combined to assess negative environmental stressors, and an additional item was incorporated to evaluate the overall perception of stress in college life. The Cronbach’s *α* for this scale was 0.907.

#### 2.4.3. Coping Style Scale

The Coping Style Scale employed in this study was the Coping Style Scale for Chinese Adults developed by B. Y. Liang and Wu (2013). This scale categorizes coping styles into two dimensions: problem-focused (19 items) and emotion-focused (14 items). Problem-focused coping styles direct attention towards stress and the problems it engenders, aiming to resolve these issues by eliminating or reducing stressors or by avoiding or escaping from them. Typical items include “Contact the parties involved in conflicts to solve the problems faced”. Emotion-focused coping styles concentrate on the emotional distress caused by stress, involving concealment, suppression, or expression of emotional pain. Typical items include “Listening to music or playing musical instruments to express personal emotions and reduce stress or pain”. The scale uses a 4-point rating scale from 1 (“almost never”) to 4 (“almost always”), with certain maladaptive items reverse-coded. In previous validation, the scale demonstrated good reliability: total Cronbach’s *α* = 0.849; 0.807 for problem-focused; and 0.720 for emotion-focused coping. Test–retest reliabilities ranged from 0.704 to 0.854, and the scale also showed sound content, structural, convergent, and criterion validity. In the present analysis, coping was treated as a composite score by calculating the mean of all items across both subscales (problem-focused and emotion-focused), representing the general tendency to adopt coping strategies. Both subscales were retained because, in the context of this scale, higher scores on either dimension (after reverse coding of maladaptive items) reflect constructive responses to stress—whether through problem-solving or emotion regulation. Thus, rather than emphasizing only one dimension, the composite score better captures the overall adaptive coping tendency. This approach aligns with the theoretical rationale of this study, which conceptualizes coping as an integrated behavioral resource in response to stressors. In this study, the Cronbach’s α for the composite score was 0.766.

#### 2.4.4. General Self-Efficacy Scale

The General Self-Efficacy Scale (GSES) used in this study was developed by Schwarzer and Jerusalem (2010) and adapted for Chinese samples by Zhang and Schwarzer. It contains 10 items rated on a 4-point scale from “not at all true” to “exactly true”. A sample item is “I can always manage to solve difficult problems if I try hard enough”. Higher scores reflect higher levels of self-efficacy. Prior research has demonstrated excellent psychometric properties, with Cronbach’s *α* ranging from 0.85 to 0.91 across Chinese populations, along with strong construct and criterion-related validity. In the present study, the Cronbach’s *α* for the GSES was 0.913.

Given that the stressors assessed in this study span multiple life domains—including academic, interpersonal, daily life, and planning-related stress—using a general measure of self-efficacy provides better conceptual alignment with the construct of Generalized Resistance Resources.

#### 2.4.5. Stress Mindset Scale

Stress mindset was measured using the Stress Mindset Measure (SMM) developed by Crum et al. (2013), which consists of two dimensions: stress-is-enhancing and stress-is-debilitating. The scale includes eight items (e.g., “Experiencing stress improves my work performance and efficiency”; “Experiencing stress depletes my energy and health”) rated on a 5-point Likert scale (0 = strongly disagree, 4 = strongly agree). Items reflecting a stress-is-debilitating mindset were reverse-scored, and higher average scores indicate a more stress-is-enhancing mindset. The SMM has demonstrated solid internal consistency (Cronbach’s *α* ranging from 0.76 to 0.86) and a stable two-factor structure in previous studies with college samples. In this study, the Cronbach’s *α* was 0.765.

### 2.5. Data Analysis

SPSS 27.0 was used to conduct reliability tests, common method bias tests, descriptive statistical analyses, and correlation analyses of the scales. The fully observed variable path model was constructed using Mplus 8.0 and calculated for chain mediation and moderated chain mediation (*p* < 0.05), respectively, using the bias-corrected nonparametric percentile bootstrap method [5000 repetitions, 95% *CI*s]. The effects were considered statistically significant if the path factor did not contain zero within the confidence interval. Gender, academic year (grade), and major were included as control variables in all structural models to account for potential confounding effects.

## 3. Results

### 3.1. Common Method Deviation Test and Collinearity Test

The Harman single-factor method was employed to assess the potential common method bias (Podsakoff et al., 2003). The unrotated factor analysis results revealed the emergence of 20 factors with eigenvalues exceeding one, and a maximum factor variance explained 18.25%, which is below the general threshold of 40%, suggesting the absence of significant common method bias in this study. A collinearity test was performed for independent, mediator, and moderator variables. The results indicated that the tolerance values for all variables exceeded 0.1, and the VIF values were below 10, indicating no serious collinearity among the variables.

### 3.2. Descriptive Statistics and Correlation Analysis

As expected, Pearson’s correlation analysis revealed significant negative correlations between stressful life events and coping styles, general self-efficacy, SOC, and stress mindset. There were significant positive correlations between coping style and general self-efficacy, stress mindset, and SOC. Additionally, there were significant positive correlations between general self-efficacy and stress mindset as well as SOC. The strength of the correlations ranged from weak (e.g., *r* = −0.261) to moderate (e.g., *r* = −0.669), suggesting modest to moderate associations among the key variables (see Table 1).

### 3.3. Mediation Test

A fully observed variable path model with moderated chain mediation was constructed using Mplus 8.0. Although the RMSEA value was above the conventional threshold (0.137), this is not uncommon in fully observed variable path models with small degrees of freedom. The model demonstrated an acceptable fit with CFI = 0.970, TLI = 0.911 (CFI, TLI > 0.90), and SRMR = 0.030 (SRMR < 0.80), and all structural paths were statistically significant. Therefore, the model structure was retained for theoretical interpretability.

**Figure 2:**
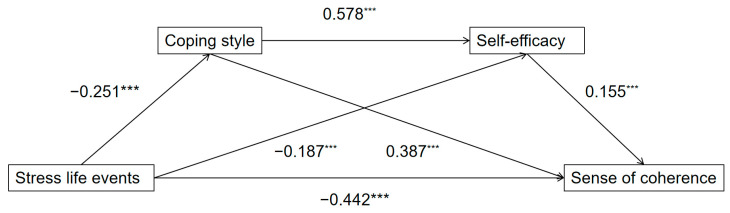
Path diagram of the effect of stressful life events on sense of coherence, reporting standardized coefficients *** *p* < 0.001.

The results (Table 2 and Figure 2) showed that stressful life events negatively predicted SOC (*β* = −0.442, *p* < 0.001), supporting Hypothesis 1. Stressful life events also negatively predicted coping styles (*β* = −0.251, *p* < 0.001) and general self-efficacy (*β* = −0.187, *p* < 0.001). Coping style positively predicted general self-efficacy (*β* = 0.578, *p* < 0.001) and SOC (*β* = 0.387, *p* < 0.001). General self-efficacy positively predicted SOC (*β* = 0.155, *p* < 0.001). The model explained 64.8% of the variance in SOC (*R*^2^ = 0.648), indicating strong predictive power.

A sample of 5000 iterations was used to test the mediating effects of the study variables, using the bias-corrected percentile bootstrap method. The results showed (Table 3) that the 95% confidence intervals of the three paths did not include “0”, indicating that the mediating effects of the three paths were significant and Hypotheses 2 and 3 were valid. The total mediation effect value was −0.148, and the relative mediating effects of the three paths were 65.54, 19.60, and 14.86% for the three paths, respectively. As the direct effect of stressful life events on SOC remained statistically significant after including the mediators (*β* = −0.442, *p* < 0.001), the mediation is best characterized as partial rather than full.

### 3.4. Moderation Test

**Figure 3:**
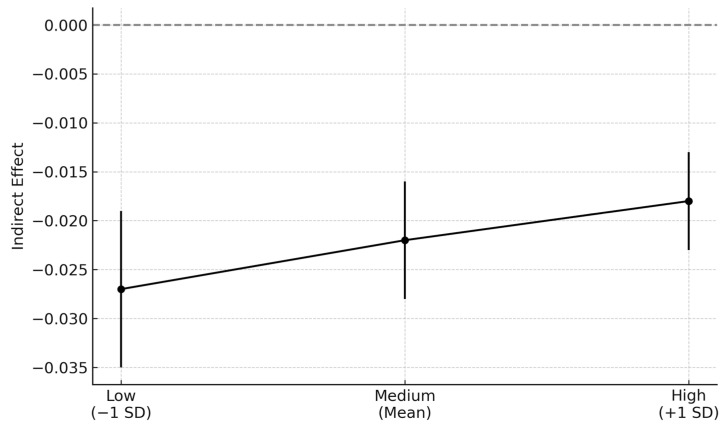
Chain mediation effect under all levels of stress mindset. This figure illustrates the conditional indirect effect of stressful life events on SOC via coping and self-efficacy at low (−1 SD), medium (mean), and high (+1 SD) levels of stress mindset. The upward trend in the line indicates that the strength of the negative indirect effect weakens as stress mindset increases, visually representing the moderating role of stress mindset.

The results showed that a stress mindset positively predicted coping style (*β* = 0.625, *SE* = 0.015, *p* < 0.001, 95%CI = [0.595, 0.655]), while the interaction term for stressful life events and stress mindset also significantly predicted coping style (*β* = 0.054, *SE* = 0.019, *p* = 0.005, 95%CI = [0.015, 0.091]). The chain mediation path was significant under all levels of stress mindset, with conditional indirect effects of −0.027 (low), −0.022 (mean), and −0.018 (high) (Table 4 and Figure 3). A pairwise contrast indicated that the difference in chain mediation effects between high and low stress mindset levels was statistically significant (Δ = 0.009, *p* = 0.007), suggesting a weakening of the negative indirect effect as the moderator increased. This suggests that individuals with a more stress-is-enhancing mindset are less negatively affected by stressful life events in terms of their coping responses, thereby reducing the strength of the indirect effect on SOC. Specifically, at the low level of stress mindset (−1 *SD*), the indirect effect of stressful life events on SOC through coping and self-efficacy was −0.027, while at the high level (+1 *SD*), the effect was −0.018. This difference of 0.009 indicates that as stress mindset becomes more enhancing, the negative association between stressors on SOC through coping-related pathways significantly weakens. As an exploratory analysis, we found that stressful life events significantly negatively predicted stress mindset (*β* = −0.270, *R*^2^ = 0.073, *p* < 0.001), suggesting that greater exposure to stressors may be associated with a more stress-is-debilitating mindset. Thus, Hypothesis 4 is supported.

## 4. Discussion

The present study investigated the association between stressful life events and college students’ sense of coherence (SOC), with a particular focus on the mediating roles of coping style and general self-efficacy as well as the moderating role of stress mindset. The results supported all four proposed hypotheses, offering insight into how cognitive and behavioral factors jointly shape SOC development. These findings corroborate all the proposed hypotheses. First, stressful life events were significantly associated with lower SOC, which is consistent with Hypothesis 1. Second, coping style appeared to mediate the relationship between stressors and SOC (supporting Hypothesis 2), and in conjunction with self-efficacy, formed a significant chain mediation (supporting Hypothesis 3). Specifically, students who encountered more stressful events tended to demonstrate less adaptive coping strategies, as reflected by lower scores on both problem-focused and emotion-focused coping dimensions. This indicates a reduced inclination to either actively resolve problems or express and manage emotional distress. These students also exhibited lower general self-efficacy, which was subsequently associated with diminished SOC. Notably, the indirect effect mediated solely through coping style accounted for the largest proportion of the total indirect effect (approximately 65%), followed by self-efficacy (about 20%), while the sequential pathway through both coping and self-efficacy, although smaller (around 15%), was also significant. Third, the results supported Hypothesis 4, demonstrating that a stress mindset moderated the relationship between stressful events and coping style. Compared with students with a “stress-is-debilitating” mindset, those with a more positive “stress-is-enhancing” mindset showed a weaker association between stressful events on coping, resulting in a smaller indirect effect of stress on SOC through reduced coping. In essence, students predisposed to view stress positively were more capable of engaging in effective coping strategies even under pressure, thereby better preserving their SOC. Although the moderation effect was statistically significant, the magnitude was modest (Δ = 0.009), which is common in psychological moderation models. Thus, its theoretical relevance should be understood as supportive rather than decisive.

These findings can be interpreted within the framework of Antonovsky’s salutogenic model (Antonovsky, 1987). According to this model, stressors, coping processes, and Generalized Resistance Resources (GRRs) collectively influence the development of the sense of coherence (SOC). Our results are consistent with this framework: stressful life events as chronic stressors tend to diminish an individual’s SOC in the absence of effective coping mechanisms. An increase in stressful events may lead to repressed or avoidant coping responses, potentially resulting from burnout and psychological resource depletion. This may impair individuals’ ability to identify and utilize appropriate coping resources in stressful situations (Super et al., 2015). The mediating role of coping style indicates that it is not merely the amount of stress experienced, but rather how one manages that stress, which determines SOC. In other words, strengthening one’s coping responses—such as actively seeking support, engaging in problem-solving, or constructively expressing emotions—can enhance SOC by facilitating a more effective mobilization of internal and external resources. This interpretation aligns with the behavioral pathway of SOC development proposed by Super et al. (2015).

The moderating effect of stress mindset observed in this study is consistent with prior research on stress mindsets. Our data showed that students with a more positive stress mindset (i.e., a “stress-is-enhancing” mindset) coped more effectively under high-stress conditions, which parallels the findings in other populations. Crum et al. (2013) and Jamieson et al. (2018) have noted that endorsing a “stress-is-enhancing” mindset can lead to better performance and promote positive coping behaviors under stress. Empirical studies have likewise confirmed this mechanism, as individuals who view stress as beneficial are more likely to engage in adaptive coping strategies (such as active coping and positive reappraisal) and are less prone to negative emotional outcomes (Keech et al., 2018; Jiang et al., 2019; Grünenwald et al., 2023). Consistent with these findings, we found that a positive stress mindset appeared to buffer the negative relationship between stressful life events and coping, which corresponded to a reduced indirect association with SOC. Our findings suggest that stress mindset may also function as an internal Generalized Resistance Resource, particularly by promoting more adaptive coping responses under stress. Rather than enhancing SOC directly, stress mindset contributes to the behavioral stage of SOC development by facilitating successful stress management through improved coping. This behavioral mechanism aligns with the salutogenic model’s emphasis on how internal resources support tension reduction via effective coping processes.

Notably, our study demonstrated a significant chain mediation effect: stress influenced SOC not only through coping style alone, but also via the sequential path “stress → coping → self-efficacy → SOC”. While numerous studies have identified a strong positive association between self-efficacy and SOC (e.g., Greco et al., 2023; Schäfer et al., 2023), relatively few have examined how coping-related experiences may contribute to self-efficacy beliefs, which in turn may support SOC development. According to self-efficacy theory, mastery experiences are a primary source of self-efficacy development (Usher & Pajares, 2009). Our findings are consistent with this theoretical proposition, suggesting that successful coping—by helping individuals perceive their capacity to manage stressors—may enhance general self-efficacy and thereby support a stronger sense of coherence. This pattern aligns with the perceptual mechanism of SOC development proposed by Super et al. (2015), wherein GRRs function by enhancing individuals’ perception of life as comprehensible, manageable, and meaningful.

While the theoretical link between coping mastery and self-efficacy is well established, our study provides empirical evidence that connects this perceptual pathway to SOC formation in a student population under stress. In particular, general self-efficacy was conceptualized as a cross-situational internal GRR and used to test its potential function within the perceptual pathway of SOC development. This represents a meaningful extension of salutogenic theory by clarifying how internal belief-based GRRs operate via perceptual mechanisms in the development of SOC.

Given that general self-efficacy reflects a broad, domain-general belief system, it is worth noting that domain-specific self-efficacy (e.g., coping self-efficacy, academic self-efficacy) might exert even stronger mediating effects in specific stress contexts. In such cases, self-efficacy beliefs aligned more closely with the nature of the stressor may serve as more proximal psychological resources, enhancing prediction accuracy. Future research could therefore explore the comparative utility of general versus domain-specific self-efficacy in SOC-related processes.

Taken together, our findings suggest that although both stress mindset and general self-efficacy can be understood as belief-based internal GRRs, they may contribute to SOC development at different stages of the adjustment process. Stress mindset was associated with the behavioral mechanism by moderating the stress–coping link, while self-efficacy was situated in the perceptual path linking coping to SOC. This positional difference, as reflected in the model structure (Antonovsky, 1979; Super et al., 2015), highlights the potential value of examining how distinct GRRs operate across different stages or components of salutogenic processes. We do not claim to have tested the full range of GRR functions, but our findings point to the importance of distinguishing how internal resources exert their effects—whether by facilitating coping behavior or by reinforcing coherence-related appraisals. Future research could build on this approach to develop a more functionally differentiated understanding of GRRs, which may ultimately inform more targeted intervention strategies to strengthen SOC.

Finally, this study adds to the growing literature suggesting that stress, when appraised positively and met with effective coping, may contribute to the development of SOC. This interpretation is consistent with Antonovsky’s (1987) proposition that stressors are not inherently harmful but can serve as potential catalysts for health and psychological resource growth, depending on how they are managed. In the salutogenic model, tension is viewed as a necessary condition for mobilizing GRRs and fostering resilience (Langeland & Vinje, 2013). Recent research on stress optimization further supports this view, showing that adaptive responses to stress—such as cognitive reappraisal and stress-is-enhancing mindsets—may promote learning, performance, and well-being (Crum et al., 2020). Among university students, moderate and manageable stress exposure, when paired with sufficient internal resources and support, may facilitate the development of psychological strengths such as self-efficacy and SOC. Therefore, rather than focusing solely on stress reduction, interventions should aim to help students reframe stress as a challenge, develop adaptive coping skills, and activate internal GRRs. This approach may be more aligned with how SOC develops over time and may provide a more sustainable foundation for mental health promotion.

## 5. Conclusions

### 5.1. General Conclusions

In conclusion, the present study provides a theory-informed framework for understanding how stressful life events may relate to college students’ sense of coherence, through the potential mediating roles of coping style and general self-efficacy, and the moderating role of stress mindset. Based on data from a large sample of Chinese university students, our proposed model was supported and aligned with the expectations of the salutogenic model. The findings suggest that the development of SOC may be shaped by the dynamic interplay of stressors, coping behaviors, personal beliefs, and internal resources. This study is among the first to incorporate stress mindset into SOC research, indicating that positive beliefs about stress may buffer its negative associations with adaptive coping. These findings contribute to the growing literature on how cognitive and behavioral processes jointly relate to SOC during the stress adaptation process.

### 5.2. Limitations and Future Directions

On a practical level, this study offers initial guidance for promoting students’ SOC by highlighting the potential benefits of interventions that strengthen adaptive coping skills, reinforce general self-efficacy, and cultivate a “stress-is-enhancing” mindset. However, several limitations should be noted.

First, the cross-sectional design prevents causal inference, and reliance on self-report measures may introduce common method bias. Future studies should adopt longitudinal or experimental approaches to further examine whether improvements in coping, self-efficacy, or stress mindset are associated with increases in SOC over time.

Second, the study relied on convenience sampling from several universities, which may limit the generalizability of the findings due to potential selection bias. All participants were Chinese university students, and the sample was predominantly male. These characteristics may influence the observed relationships and limit their applicability to other cultural contexts or more gender-balanced populations. Future research should aim to replicate this model in more diverse settings.

Third, this study did not account for individuals’ early adversity experiences (e.g., adverse childhood experiences), which may play a role in shaping internal resources and stress responses. Future research could examine whether such developmental factors moderate the stress–SOC pathways identified here.

Finally, future studies may consider testing additional mediators or moderators beyond those investigated in this study. Variables such as social support, emotional regulation, or academic pressure may provide a more comprehensive picture of how SOC develops in stressful contexts and reveal new points for intervention.

Despite these limitations, our findings offer preliminary support for extending the salutogenic model by illustrating how stress-related experiences may relate to SOC through both behavioral and perceptual mechanisms. By identifying modifiable psychological factors linked to SOC, this study contributes to the advancement of strength-based approaches to student mental health promotion.

## Figures and Tables

**Table 1:** Descriptive statistics between variables and results of correlation analysis between variables.

Variables	*M*(*SD*)	1	2	3	4	5	6	7	8
1. Gender ^a^	1.36(0.48)	1							
2. Grade ^b^	1.67(0.87)	0.024	1						
3. Major ^c^	1.81(0.59)	−0.296 ***	0.087 *	1					
4. Stressful life events	2.44(0.61)	0.066 **	0.107 ***	0.028	1				
5. Stress mindset	3.28(0.66)	−0.008	−0.004	−0.099 **	−0.261 ***	1			
6. Coping style	2.60(0.29)	0.004	0.018	−0.078 *	−0.418 ***	0.618 ***	1		
7. Self-efficacy	2.48(0.59)	−0.046 *	0.052 **	−0.075 *	−0.428 ***	0.458 ***	0.650 ***	1	
8. Sense of coherence	4.45(0.86)	−0.115 ***	−0.061 **	−0.081 *	−0.669 ***	0.524 ***	0.666 ***	0.597 ***	1

Note: * *p* < 0.05; ** *p* < 0.01; *** *p* < 0.001. ^a^ 1 = male; 2 = female; ^b^ 1 = freshmen; 2 = sophomores; 3 = juniors; 4 = seniors. ^c^ 1 = science and engineering; 2 = humanities and social sciences; 3 = others.

**Table 2:** Regression analysis of variable relationships in the mediation model.

Regression Equations		Significance of Regression Coefficient		95% CI	
Outcome Variables	Predictor Variables	*β*	*SE*	LLCT	ULCT
Coping style	Stressful life events	−0.251 ***	0.015	−0.281	−0.221
Self-efficacy	Stressful life events	−0.187 ***	0.017	−0.219	−0.152
	Coping style	0.578 ***	0.015	0.547	0.608
Sense of coherence	Stressful life events	−0.442 ***	0.016	−0.472	−0.411
	Coping style	0.387 ***	0.020	0.348	0.424
	Self-efficacy	0.155 ***	0.018	0.119	0.190

Note: Standardized regression coefficients are reported; *** *p* < 0.001.

**Table 3:** Analysis of the mediating effect of sense of coherence and stressful life events.

Trails	Effect	Relative Mediation Effect	BootSE	Boot LLCT	Boot ULCT
Total mediating effects: Ind1+Ind2+Ind3	−0.148 ^a^				
Ind1: stressful life events→coping style→SOC	−0.097 ^a^	65.54%	0.008	−0.112	−0.083
Ind2: stressful life events→self-efficacy→SOC	−0.029 ^a^	19.60%	0.004	−0.038	−0.021
Ind3: stressful life events→coping style→self-efficacy→SOC	−0.022 ^a^	14.86%	0.003	−0.029	−0.016

Note: Non-standardized regression coefficients are reported and ‘^a^’ denotes a significant effect.

**Table 4:** Moderating effect of stress mindset on the chain mediation path.

Path	Conditional Indirect	*B*	*SE*	95%*CI*	*p*
Stressful life events → coping style → self-efficacy → SOC	Stress mindset (low)	−0.027	0.004	[−0.035, −0.019]	<0.001
	Stress mindset (middle)	−0.022	0.003	[−0.028, −0.016]	<0.001
	Stress mindset (high)	−0.018	0.003	[−0.023, −0.013]	<0.001
	Contrast (high vs. low)	0.009	0.003	[0.003, 0.016]	0.007

Note: Non-standardized regression coefficients are reported.

## Data Availability

Raw data supporting the conclusions of this article will be made available by the authors without undue reservation.

## References

[B1-behavsci-15-00762] Antonovsky A. (1979). Health, stress and coping.

[B2-behavsci-15-00762] Antonovsky A. (1987). Unraveling the mystery of health: How people manage stress and stay well.

[B3-behavsci-15-00762] Bachem R., Makhashvili N., Maercker A., Javakhishvili J. D., Aeschlimann A., Pilauri K., Latibashvili T., Levin Y., Shengelia N. (2023). University students’ life stressors and mental health in Georgia and German-speaking Switzerland: Exploring the role of fatalism, sense of coherence, cross-cultural coping, and help-seeking. International Perspectives in Psychology: Research, Practice, Consultation.

[B4-behavsci-15-00762] Bao L. P., Liu J. S. (2005). The reliability and validity of Chinese version of SOC-13. Chinese Journal of Clinical Psychology.

[B5-behavsci-15-00762] Braun-Lewensohn O., Idan O., Lindstrom B., Margalit M. (2022). Salutogenesis and the sense of coherence during the adolescent years. The handbook of salutogenesis.

[B6-behavsci-15-00762] Braun-Lewensohn O., Sagy S. (2010). Sense of coherence, hope and values among adolescents under missile attacks: A longitudinal study. International Journal of Children’s Spirituality.

[B7-behavsci-15-00762] Braun-Lewensohn O., Sagy S., Sabato H., Galili R. (2013). Sense of coherence and sense of community as coping resources of religious adolescents before and after the disengagement* from the Gaza strip. The Israel Annals of Psychiatry and Related Disciplines.

[B8-behavsci-15-00762] Chen J. W., Wang T. (2012). The college students’ stressful events, emotional reaction and coping styles. Journal of Higher Education.

[B9-behavsci-15-00762] Chen L., Qu L. (2021). From stressful experiences to depression in Chinese migrant children: The roles of stress mindset and coping. Frontiers in Psychology.

[B10-behavsci-15-00762] Crum A. J., Akinola M., Martin A., Fath S. (2017). The role of stress mindset in shaping cognitive, emotional, and physiological responses to challenging and threatening stress. Anxiety, Stress, and Coping.

[B11-behavsci-15-00762] Crum A. J., Jamieson J. P., Akinola M. (2020). Optimizing stress: An integrated intervention for regulating stress responses. Emotion.

[B12-behavsci-15-00762] Crum A. J., Salovey P., Achor S. (2013). Rethinking stress: The role of mindsets in determining the stress response. Journal of Personality and Social Psychology.

[B13-behavsci-15-00762] Eriksson M. (2005). Validity of Antonovsky’s sense of coherence scale: A systematic review. Journal of Epidemiology & Community Health.

[B14-behavsci-15-00762] Eriksson M., Wennerberg M., Lundgren S., Danielson E. (2017). “Self-employed” in caregivinghood: The contribution of Swedish informal caregivers’ environmental and contextual resistance resources and deficits. Societies.

[B15-behavsci-15-00762] Fagermoen M. S., Hamilton G., Lerdal A. (2015). Morbid obese adults increased their sense of coherence 1 year after a patient education course: A longitudinal study. Journal of Multidisciplinary Healthcare.

[B16-behavsci-15-00762] Greco A., Adorni R., De Matteis C., D’Addario M., Fattirolli F., Franzelli C., Giannattasio C., Luyckx K., Steca P. (2023). Latent change models of lifestyle in acute coronary syndrome patients: Are lifestyle changes associated with resilience changes?. Health Psychology Open.

[B17-behavsci-15-00762] Grünenwald I., Kaluza A. J., Schultze M., van Dick R. (2023). Stress mindset and social identification in chronic pain patients and their relationship to coping, well-being & depression. Journal of Clinical Psychology in Medical Settings.

[B18-behavsci-15-00762] He Y., Zhang Z., Yang X. H. (2023). A study on relationship among perceived stress, positive coping style and life satisfaction of college students. Journal of Shangluo University.

[B19-behavsci-15-00762] Jamieson J. P., Crum A. J., Goyer J. P., Marotta M. E., Akinola M. (2018). Optimizing stress responses with reappraisal and mindset interventions: An integrated model. Grantee Submission, Anxiety.

[B20-behavsci-15-00762] Jiang Y., Zhang J., Ming H., Huang S., Lin D. (2019). Stressful life events and well-being among rural-to-urban migrant adolescents: The moderating role of the stress mindset and differences between genders. Journal of Adolescence.

[B21-behavsci-15-00762] Kähönen K., Näätänen P., Tolvanen A., Salmela-Aro K. (2012). Development of sense of coherence during two group interventions. Scandinavian Journal of Psychology.

[B22-behavsci-15-00762] Keech J. J., Hagger M. S., O’Callaghan F. V., Hamilton K. (2018). The influence of university students’ stress mindsets on health and performance outcomes. Annals of Behavioral Medicine: A Publication of the Society of Behavioral Medicine.

[B23-behavsci-15-00762] Kulcar V., Kreh A., Juen B., Siller H. (2023). The role of sense of coherence during the COVID-19 crisis: Does it exercise a moderating or a mediating effect on university students’ wellbeing?. SAGE Open.

[B24-behavsci-15-00762] Kunzler A. M., Helmreich I., Chmitorz A., König J., Binder H., Wessa M., Lieb K. (2020). Psychological interventions to foster resilience in healthcare professionals. Cochrane Database of Systematic Reviews.

[B25-behavsci-15-00762] Langeland E., Vinje H. F., Keyes C. L. M. (2013). The significance of salutogenesis and well-being in mental health promotion: From theory to practice. Mental well-being: International contributions to the study of positive mental health.

[B26-behavsci-15-00762] Lazarus R. S. (1993). Coping theory and research: Past, present, and future. Psychosomatic Medicine.

[B27-behavsci-15-00762] Lazarus R. S., Folkman S. (1984). Stress, appraisal, and coping.

[B28-behavsci-15-00762] Liang B. Y., Wu Y. C. (2013). Psychological health diathesis assessment system: The development of coping style scale for Chinese adults. Studies of Psychology and Behavior.

[B29-behavsci-15-00762] Liang H. Y., Li F., Wang Q. Y., Chen S. (2015). Gratitude and sense of coherence among high school students: Mediating roles of positive affect and positive coping style. Journal of Psychological Science.

[B30-behavsci-15-00762] Lindahl M., Juneja H., Teljigovic S., Rafn J., Nielsen N. O. (2021). Change in sense of coherence and health-related quality of life after injury—A prospective cohort study. Disability & Rehabilitation.

[B31-behavsci-15-00762] Liu J. S., Zhou Y., Bao L. P., Sang B. (2006). The relationship between sense of coherence and coping style among adolescents. Psychological Science.

[B32-behavsci-15-00762] Maass R., Lindström B., Lillefjell M. (2017). Neighborhood-resources for the development of a strong SOC and the importance of understanding why and how resources work: A grounded theory approach. BMC Public Health.

[B33-behavsci-15-00762] Mato M., Tsukasaki K. (2019). Factors promoting sense of coherence among university students in urban areas of Japan: Individual-level social capital, self-efficacy, and mental health. Global Health Promotion.

[B34-behavsci-15-00762] Peer N., Lombard C., Steyn K., Levitt N. (2020). A high burden of adverse life events and poor coping mechanisms experienced by urban-dwelling black South Africans. PLoS ONE.

[B35-behavsci-15-00762] Podsakoff P. M., MacKenzie S. B., Lee J.-Y., Podsakoff N. P. (2003). Common method biases in behavioral research: A critical review of the literature and recommended remedies. The Journal of Applied Psychology.

[B36-behavsci-15-00762] Sanna F., Galletta M., Koelen M., Contu P. (2022). Development of sense of coherence stability in the AGORA healthy ageing study. International Journal of Environmental Research and Public Health.

[B37-behavsci-15-00762] Schäfer S. K., Sopp M. R., Fuchs A., Kotzur M., Maahs L., Michael T. (2023). The relationship between sense of coherence and mental health problems from childhood to young adulthood: A meta-analysis. Journal of Affective Disorders.

[B38-behavsci-15-00762] Schäfer S. K., Sopp M. R., Koch M., Göritz A. S., Michael T. (2022). The long-term buffering effect of sense of coherence on psychopathological symptoms during the first year of the COVID-19 pandemic: A prospective observational study. Journal of Psychiatric Research.

[B39-behavsci-15-00762] Schwarzer R., Born A. (1997). Optimistic self-beliefs: Assessment of general perceived self-efficacy in thirteen cultures. World Psychology.

[B40-behavsci-15-00762] Schwarzer R., Jerusalem M. (2010). The general self-efficacy scale (GSE). Anxiety, Stress, and Coping.

[B41-behavsci-15-00762] Super S., Wagemakers M. A. E., Picavet H. S. J., Verkooijen K. T., Koelen M. A. (2015). Strengthening sense of coherence: Opportunities for theory building in health promotion. Health Promotion International.

[B42-behavsci-15-00762] Tsuno Y. S., Yamazaki Y. (2007). A comparative study of Sense of Coherence (SOC) and related psychosocial factors among urban versus rural residents in Japan. Personality and Individual Differences.

[B43-behavsci-15-00762] Usher E. L., Pajares F. (2009). Sources of self-efficacy in mathematics: A validation study. Contemporary Educational Psychology.

[B44-behavsci-15-00762] Veronese G., Mahamid F. A., Bdier D. (2022). Subjective well-being, sense of coherence, and posttraumatic growth mediate the association between COVID-19 stress, trauma, and burnout among Palestinian health-care providers. American Journal of Orthopsychiatry.

[B45-behavsci-15-00762] Yang H. (2015). Research on resilience process: Stressful events, self-discrepancy, social support, and actively coping style as predictors, school adjustment as dependent variable. Master’s thesis.

[B46-behavsci-15-00762] Yeager D. S., Dweck C. S. (2012). Mindsets that promote resilience: When students believe that personal characteristics can be developed. Educational Psychologist.

[B47-behavsci-15-00762] Yuan S., Chen Y. G., Huang X. T. (2023). Effects of stressful life events, life attitude and coping strategy on depression of college students. Community Psychology Research.

[B48-behavsci-15-00762] Yuan Y. Y., Wu M. X., Wang Z. H., Li Z. H. (2020). Family socioeconomic status and children’s general self-efficacy: Chain mediation of parents’ care and coping style. Chinese Journal of Clinical Psychology.

[B49-behavsci-15-00762] Zeng J., Shi H. Y., Zhang H. (2016). The structure of sense of coherence and its influencing factors. Journal of South West University (Social Sciences Edition).

